# Postoperative complications and mobilisation following major abdominal surgery with vs. without fitness tracker-based feedback (EXPELLIARMUS): study protocol for a student-led multicentre randomised controlled trial (CHIR-*Net* SIGMA study group)

**DOI:** 10.1186/s13063-020-4220-8

**Published:** 2020-03-23

**Authors:** Marius Schwab, Niall Brindl, Alexander Studier-Fischer, Thomas Tu, Julia Gsenger, Max Pilgrim, Mirco Friedrich, Pia-Elena Frey, Christina Achilles, Alexander Leuck, Thore Bürgel, Manuel Feisst, Christina Klose, Solveig Tenckhoff, Colette Dörr-Harim, André L. Mihaljevic

**Affiliations:** 1 Faculty of Medicine, University of Heidelberg, Im Neuenheimer Feld 346, 69120 Heidelberg, Germany; 2 Health Data Science Unit, University Hospital Heidelberg, BioQuant, Im Neuenheimer Feld 267, 69120 Heidelberg, Germany; 3 Institute of Medical Biometry and Informatics, University Hospital Heidelberg, Im Neuenheimer Feld 130.3, 69120 Heidelberg, Germany; 4 CHIR-Net Coordination Centre at the Study Centre of the German Surgical Society (SDGC), University Hospital Heidelberg, Im Neuenheimer Feld 130.3, 69120 Heidelberg, Germany; 5 Department of General, Visceral and Transplantation Surgery, University Hospital Heidelberg, Im Neuenheimer Feld 110, 69120 Heidelberg, Germany

**Keywords:** Wearable fitness trackers, Postoperative complications, Major abdominal surgery, Quality of life, Recovery of function, Comprehensive Complication Index (CCI), Mobilisation, Postoperative outcomes, Dindo-Clavien, Randomised controlled trial

## Abstract

**Background:**

Postoperative complications following major abdominal surgery are frequent despite progress in surgical technique and perioperative care. Early and enhanced postoperative mobilisation has been advocated to reduce postoperative complications, but it is still unknown whether it can independently improve outcomes after major surgery. Fitness trackers (FTs) are a promising tool to improve postoperative mobilisation, but their effect on postoperative complications and recovery has not been investigated in clinical trials.

**Methods:**

This is a multicentre randomised controlled trial with two parallel study groups evaluating the efficacy of an enhanced and early mobilisation protocol in combination with FT-based feedback in patients undergoing elective major abdominal surgery. Participants are randomly assigned (1:1) to either the experimental group, which receives daily step goals and a FT giving feedback about daily steps, or the control group, which is mobilised according to hospital standards. The control group also receives a FT, however with a blackened screen; thus no FT-based feedback is possible. Randomisation will be stratified by type of surgery (laparoscopic vs. open). The primary endpoint of the study is postoperative morbidity within 30 days measured via the Comprehensive Complication Index. Secondary endpoints include number of steps as well as a set of functional, morbidity and safety parameters. A total of 348 patients will be recruited in 15 German centres. The study will be conducted and organised by the student-led German Clinical Trial Network SIGMA.

**Discussion:**

Our study aims at investigating whether the implementation of a simple mobilisation protocol in combination with FT-based feedback can reduce postoperative morbidity in patients undergoing major abdominal surgery. If so, FTs would offer a cost-effective intervention to enhance postoperative mobilisation and improve patient outcomes.

**Trial registration:**

Deutsches Register Klinischer Studien (DRKS, German Clinical Trials Register): DRKS00016755, UTN U1111-1228-3320. Registered on 06.03.2019.

## Background

Postoperative morbidity, mortality and patient-reported outcomes are arguably the most relevant outcome parameters in surgery, enabling a full risk-benefit assessment of surgical interventions. Although mortality for major abdominal surgery has decreased significantly over the last decades, morbidity remains high [1–4]. Complication rates between 30 and 60% have repeatedly been reported in randomised controlled trials (RCTs) for pancreatic [5–8], hepatobiliary [9], colorectal [10–12] and upper gastrointestinal surgery [13–15]. Interestingly, non-surgical complications like pulmonary and cardiac complications constitute a substantial part of overall morbidity [13].

Postoperative ambulation/mobilisation has been postulated to decrease postoperative complications in abdominal surgery and is part of all Enhanced Recovery After Surgery (ERAS) guidelines [16, 17]. However, the quality of the evidence to support this recommendation is very low [18–21]. Therefore, it is still unknown whether early and enhanced mobilisation can independently improve outcomes after major abdominal surgery and, if so, how this is best implemented in daily clinical practice [22].

Although designed as a consumer product to help motivate individuals to be physically active, wearable fitness trackers (FTs) are becoming increasingly popular as measurement tools in clinical research [23]. Importantly, FTs can be used to objectively measure outcomes like steps or distance covered and can thus be used as a feedback tool to meet prespecified mobilisation targets through feedback via the FT display. Few trials have studied FTs in the postoperative setting, and none used standardised assessment of postoperative complications as a primary outcome measure [24–29].

### Rationale for the trial

The high rate of postoperative complications following major abdominal surgery warrants clinical trials that investigate interventions which could potentially reduce postoperative morbidity. Early and enhanced postoperative mobilisation has been advocated to reduce postoperative complications, but it is still unknown whether it can independently improve outcomes after major abdominal surgery. FTs are a promising tool to improve postoperative mobilisation following major abdominal surgery, but their effect on postoperative complications and recovery has not been studied in high-quality clinical trials.

### Study aims and objectives

The objective of this study is to determine whether daily step goals and feedback via a FT reduces the rate of postoperative complications following elective major abdominal surgery.

### Student-led clinical research

The teaching of clinical research and its associated scientific methodology is underdeveloped in medical schools, leading to an expected deficit in academic faculty across Europe [30, 31]. Research-based learning is a concept that refers to a trend in higher education: to provide students with the opportunity to gain knowledge by conducting their own scientific inquiries or investigations [32]. In 2017 the study network of the German Surgical Society (CHIR-*Net* [33];) founded a student-led clinical trial network across Germany (SIGMA; Student-Initiated German Medical Audit, [34]) [35, 36]. SIGMA has performed an observational study investigating patient-reported outcome measures and complications after surgery [37] and will perform the EXPELLIARMUS trial. To our knowledge, this is the first multicentre RCT ever to be performed by medical students.

## Methods/design

### Trial design

EXPELLIARMUS is a multicentre RCT with two parallel study groups.

### Requirements for participating centres

Participating trial centres require at least one investigator (surgeon) and at least two medical students who are acquainted with the trial protocol and electronic case report form (eCRF). Furthermore, approval from the local ethics committee according to respective rules and regulations is mandatory.

### Recruitment of study sites

Fifteen regional centres of the clinical trial network (CHIR-*Net*, [33]) have committed to participate in the EXPELLIARMUS trial; all are university hospitals or tertiary care centres in Germany.

### Participants

#### Preoperative inclusion criteria

Participants must fulfil the following criteria:

1. Patients scheduled for elective major abdominal surgery defined as procedures expected to last more than 2 h, or with an anticipated blood loss greater than 500 ml [38]
2. Ability to understand the character and individual consequences of the clinical trial
3. Open or laparoscopic or robotic surgery or any variant (laparoscopic-assisted, hybrid procedures, etc.)
4. Provision of written informed consent
5. Age ≥ 18 years.

#### Preoperative exclusion criteria

The preoperative exclusion criteria are as follows:

1. American Society of Anesthesiologists (ASA) grade > 3
2. Preoperative immobility or inability to walk unaided
3. Participation in another interventional trial with interference of intervention and outcome of this study
4. Expected postoperative stay in the intensive care or intermediate care ward ≥ 4 days
5. Planned re-operation within 30 days after index operation
6. Planned abdominal-thoracic operations (two-field surgeries).

#### Intra/postoperative inclusion criteria

The intra/postoperative inclusion criteria are as follows:

1. Expected postoperative stay on intensive care or intermediate care ward of less than 4 days
2. No planned re-operation within 30 days
3. Confirmed major abdominal surgery (as defined in “Preoperative inclusion criteria”).

### Intervention

In the experimental group, patients are fitted with a wearable FT (ActiGraph GT9X Link, ActiGraph, ProCare, Groningen, the Netherlands) on their dominant wrist after the operation for the duration of their postoperative stay until discharge or a maximum of 30 days. If the FT cannot be placed on the dominant wrist, it should be fitted on the non-dominant wrist. If this is also not possible, the FT should be fitted to any part of the patient’s body with a clip. Patients receive real-time visual feedback via the display of their tracker regarding daily steps taken and are encouraged to meet predefined daily step goals. In addition, ambulation is encouraged by the interprofessional care teams to meet predefined step goals.

The predefined step goals/instructions (mobilisation protocol) are as follows:

1. “Please ambulate/mobilise as much as possible and allowed by your doctors”
2. “Please take more steps than yesterday”
3. “Your daily step goal should be 4000 steps per day or more. Don’t worry if you do not reach this goal immediately”
4. “You should reach this daily 4000-step target latest on postoperative day 5 in the case of laparoscopic surgery or on postoperative day 8 in the case of open surgery”.

In the control group, patients are fitted with a wearable fitness tracker (ActiGraph GT9X Link, ActiGraph, ProCare) on their dominant wrist for the duration of their postoperative stay until discharge or a maximum of 30 days. The display of the device is disabled (blackened), and accordingly no feedback via the device is given. Patients are allowed to mobilise at will, i.e. as tolerated. If possible, mobilisation is performed according to ERAS guidelines on the morning of postoperative day 1 (POD 1) or according to in-house regimes [18, 19, 21]. Ambulation is encouraged by the interprofessional care teams according to local standards (current practice), but no specific mobilisation protocol is provided.

### Assignment of intervention and randomisation

In order to achieve comparable study groups, patients will be randomly assigned to one of the two groups using a centralised web-based tool (www.randomizer.at). The online randomisation procedure provides information regarding the group allocation. Block randomisation with variable block sizes is used. Randomisation will be stratified by type of surgery (laparoscopic vs. open). Laparoscopic-assisted or hybrid procedures are counted as laparoscopic surgeries. Conversions from laparoscopic to open surgery are counted as open procedures. Randomisation will be performed on the day of surgery (visit 2) at the time of skin closure or later. Randomisation will be carried out only if all intraoperative inclusion criteria are fulfilled (see above). The online randomisation tool ensures concealment of the randomisation schedule. It is a computer-generated, concealed schedule that is not accessible by any trial participant or investigator. Randomisation will only be done by authorised trial personnel (investigator, medical student or designated representative) with their login data. Patients are recruited to the trial at least 24 h before surgery.

### Blinding

Neither patients nor outcome assessors will be blinded to the intervention, as this is unfeasible and contradicts the pragmatic character of the trial. However, given the objective nature of the primary and most secondary endpoints, the risk of bias is limited.

### Withdrawal

Patients may decide to withdraw from the study at any time without providing any specific reason for their decision. If, in the investigator’s opinion, continuation of the trial intervention would be detrimental to the subject’s well-being, the FT-based feedback will be discontinued (screen blackened). In addition, the feedback by the interprofessional care team will be discontinued, and the patient will not receive any more step goals. In both cases, the reason for withdrawal must be recorded in the CRF and in the patient’s medical records. If patient enrolment is unsatisfactory with respect to quality or quantity, or data recording is severely inaccurate or incomplete, as well as if externally respective evidence is given, the trial may be prematurely closed by the coordinating investigator in consultation with the responsible statistician and the CHIR-*Net* SIGMA steering committee. If the FT is removed, breaks or is otherwise unavailable, the clinical investigators will seek to replace it as soon as possible to guarantee complete step counts.

### Outcome measures

The primary endpoint of the study is defined as postoperative morbidity measured via the Comprehensive Complication Index (CCI) [2, 3] within 30 days after the index operation. The CCI is calculated for an individual patient based on the assessment of all complications according to the Dindo-Clavien classification [39, 40]. This classification grades postoperative complications according to their sequelae and has gained universal acceptance. Although it allows for an objective assessment of complications, it provides neither definitions of complications nor a measure of the entire impact of complications for an individual patient. The latter is addressed by the CCI, which measures the overall morbidity for an individual patient on a scale from 0 (no complication) to 100 (death). The CCI summarises all postoperative complications instead of focusing on specific complications and is thus an objective measure to assess the full burden and impact of complications in a single patient [39, 40].

The following secondary endpoints will be assessed in the EXPELLIARMUS trial:

1. Number of steps for each POD until POD 8 or until discharge, whichever comes first. This serves as a measure of success for the trial intervention.
2. Patient-completed quality of recovery assessment according to Quality of Recovery 15 (QoR-15) at baseline and on POD 4 (or at discharge, whichever comes first) [41, 42]. QoR-15 is a validated short-term measure of postoperative recovery with 15 questions [41]; it has been developed from the longer QoR-40 [42]. It covers some, but not all aspects of postoperative recovery that are important to patients and experts [38]. As for the QoR-40, it can be assumed that QoR-15 results normalise within a week after major abdominal surgery [43]; thus, assessment at baseline and on POD 4 seems reasonable to elucidate potential differences between the two groups.
3. Activity data (via the wearable device for each POD until discharge or a maximum of 30 days):
  - Metabolic equivalents of tasks (METs) rates
  - Energy expenditure
  - Raw acceleration
  - Activity and sedentary bouts
  - Physical activity intensity
  - Total sleep time.

These data are provided by the FT used in the EXPELLIARMUS trial and allow correlating activity data with clinical outcomes for exploratory analyses.

4. Health-related quality of life (HRQoL) measured via the European Organisation for the Research and Treatment of Cancer Quality of Life Questionnaire C30 (EORTC QLQ-C30) at baseline, on POD 8 (or at discharge, whichever comes first) and on POD 30 [44]. Although the HRQoL measure EORTC QLQ-C30 has been developed and validated for patients with cancer, it is the most comprehensive tool to cover aspects of postoperative recovery that are important to patients and experts [38]. Importantly, it elucidates aspects of postoperative recovery that are not covered by the QoR-15 [38].
5. Six-min walking test (6-MWT) on POD 6 (or at discharge, whichever comes first). The 6-MWT is a well-established, widely used performance-based functional test. Patients walk for 6 min along a long, flat, straight enclosed corridor with a hard surface supervised by a medical student or investigator. The test is performed according to recommendations by the American Thoracic Society [45]. Functional tests are an important aspect of postoperative recovery that are infrequently analysed in mobilisation trials [22].
6. Time (in days) until return of bowel function measured via the gastrointestinal-2 (GI-2) score defined as “The patient has tolerated solid intake (no vomiting) for 24 hours AND has passed stool” [46]. The GI-2 score is a widely used and well-established measure for the return of postoperative bowel function [46]. This assessment is completed by the patient.
7. Postoperative pulmonary complications according to the Melbourne group score [13] during hospital stay.
8. Deep vein thrombosis until POD 30. Diagnosis needs to be based on imaging results.
9. Pulmonary embolism until POD 30 (based on imaging results and/or clinical diagnosis).
10. Time in days from date of index operation to achieve uninterrupted ambulation greater than 10 min. These data will be extracted from the FT device. Similar to the 6-MWT, this is a measure of functional outcome that has been used in prior clinical trials [47].
11. Thirty-day mortality. Mortality is an important outcome measure for any surgical clinical trial. Postoperative mortality is equivalent to a grade V complication in the Dindo-Clavien classification.
12. Length of hospital stay in days (from day of surgery until day of discharge after index operation).
13. Discharge destination from the acute hospital ward (home, rehabilitation facility, nursing home or other hospital). This is an important health-care system parameter and an indirect indicator of patient fitness.
14. Pain scores according to the numeric rating scale (NRS) on PODs 2, 4 and 6 at rest and during movement. Pain will be measured using an established patient-reported outcome measure, the NRS (0-no pain to 10-worst possible pain). Furthermore, pain is one of the main barriers to adequate postoperative mobilisation [48]. Finally, increased pain could hypothetically be associated with the intervention (enhanced mobilisation) and will thus be analysed in our trial.
15. Postoperative unintended falls/collapses until day of discharge. An increased rate of intended postoperative falls/collapses could potentially be associated with the intervention and will thus be assessed as a safety measure. Clinical consequences of the falls/collapses will be accounted for by the primary endpoint (complications according to the Dindo-Clavien classification). The number is obtained by asking the treating interprofessional care team (nurses, doctors) and the patients themselves.

### Patient time line

All consecutive patients are screened preoperatively and are enlisted in a screening list. Reasons for non-enrolment must be stated. Patients are enrolled given their ability to understand the extent and nature of the trial, their written informed consent after detailed patient information and fulfilment of all preoperative inclusion and exclusion criteria. Baseline data are collected during screening/baseline visit. Surgical data are collected during visit 2. Primary and secondary outcome parameters are collected during visits 3–8 within 30 PODs. Table 1 gives an overview of data items and activities for each trial visit.

**Table 1:** Trial visits, data items and activities of the EXPELLIARMUS trial

Activity	Visit 1 (screening, enrolment)	Visit 2 (surgery, POD 0)	Visits 3–6 PODs 2, 4, 6, 8 (respective visits are omitted if patient has been discharged before)	Visit 7 (at discharge, ± 1 day)	Visit 8 (POD 30 ± 4; end of study or premature study termination)
Data items					
Demographics and baseline data	X				
HRQoL (EORTC QLQ-C30)	X		X (only POD 8 = visit 6)		X
Quality of recovery (QoR-15)	X		X (only POD 4 = visit 4)		
Surgical and anaesthesiological data		X			
Assessment of postoperative complications (according to Dindo-Clavien)			X	X	X
Assessment of re-operation			X	X	X
Assessment of bowel function^a^			X	X (only if no bowel function during previous visits)	
Assessment of postoperative pulmonary complications^b^			X	X	X
Assessment of pulmonary embolism			X	X	X
Assessment of deep vein thrombosis			X	X	X
Assessment of pain (NRS)			X	X	
Unintended fall/collapse			X	X	
Assessment of nasogastric tube, drains, urinary catheters			X (until removed)	X (until removed)	X
Assessment of physiotherapy and assisted mobilisation			X	X	X (only if patient is still in hospital)
Assessment of mobilisation target (step goal)			X	X	X (only if patient is still in hospital)
Length of hospital stay				X	X^c^
Discharge destination				X	
Activity items					
Physical activity (DASI)^d^	X				
Instruction FT	X				
Contact information for later visits	X				
Randomisation (postoperative)		X			
Attachment of FT		X			
6-MWT			X (only POD 6 = visit 5)		
Communication of step goals in the interventional group			X		
Assessment of FT (e.g. battery)			X		
Collection of FT and data transfer				X	X^c^

*6-MWT* 6-min walking test, *DASI* Duke Activity Status Index, *EORTC QLQ-C30* European Organisation for the Research and Treatment of Cancer Quality of Life Questionnaire C30, *FT* fitness tracker, *NRS* numeric rating scale, *HRQoL* health-related quality of life, *QoR* quality of recovery, *POD* postoperative day

^a^Via the GI-2 score defined as “The patient has tolerated solid intake (no vomiting) for 24 h AND has passed stool” [46]

^b^Via the Melbourne group score [13]

^c^If patient has not been discharged since index surgery

^d^Via the Duke Activity Status Index [49]

### Visit 1 (preoperative, informed consent)

All consecutive patients are screened for potential inclusion. Eligible patients are asked for informed consent. Enrolled patients are instructed on how to use the wearable FT (see “Intervention”). In addition, the following data items will be collected: (1) demographic data; (2) baseline data; (3) medical history/comorbidities; (4) HRQoL (EORTC QLQ-C30) [44]; (5) baseline functional data via the QoR-15 questionnaire [41, 42]; (6) status of physical activity via the Duke Activity Status Index (DASI) [49].

### Visit 2 (day of surgery, postoperative day 0)

On the day of surgery, data concerning the performed procedure are collected, including duration of surgery, blood loss, type of surgery and type of surgical access (open vs. laparoscopic). Furthermore, data regarding number and types of abdominal drains, nasogastric tubes, urinary catheters and postoperative analgesic are collected. At this time, randomisation will be performed (see above) and the wearable FT is attached.

### Visits 3–6 (postoperative days 2, 4, 6 and 8 (± 1 day))

Visits 3–6 have equivalent contents with the exception of the QoR-15 questionnaire, which is only administered at visit 4, the 6-MWT, which is performed only at visit 5 (or at discharge, whichever comes first) and HRQoL assessment (EORTC QLQ-C30), which is only performed at visit 6. If the patient is discharged before undergoing visits 3–6, the respective visits are skipped and the discharge visit (visit 7) is performed.

Visits 3–6 include assessment of postoperative complications according to Dindo-Clavien (primary endpoint). In addition, all secondary endpoints will be assessed. Furthermore, data on physiotherapy and mobilisation support by health-care team members other than physiotherapists (e.g. nursing staff) are collected. Finally, patients in the experimental group receive instructions about their step goals, and it is assessed whether these mobilisation targets have been met since the last visit.

### Visit 7 (day of discharge, ± 1 day)

On the day of discharge, the FTs are collected, and the data are read out and transferred over a secure upload server. Furthermore, all data items of visits 3–6 will be assessed. Finally, the discharge destination from the hospital needs to be documented. If discharge occurs before visit 5, the 6-MWT test needs to be performed at visit 7.

### Visit 8 (postoperative day 30 (± 4 days) or premature study termination)

If the patient has not been discharged, a clinical visit will be performed. In case of prior discharge, either a telephone visit can be performed or the patient can be seen in the outpatient clinic. Assessments include complications and re-operations, pulmonary complications, pulmonary embolism or deep vein thrombosis, duration of drain placements, the health-related quality of life questionnaire EORTC QLQ-C30 and data on potential physiotherapy. If the patient is still in hospital, the FTs are collected, and the data are read out and transferred over a secure upload server.

### Sample size calculation

The sample size calculation is based on the primary endpoint “CCI within 30 days after the index operation”. Assumptions are based on the literature [39, 40, 50]: a decrease of the CCI by 10 points is considered relevant by patients and clinicians (minimal important difference, [40]) and a conservative standard deviation of 20 is assumed. The CCI in major abdominal surgery (control intervention) is assumed to be 22 [39, 40]. We hypothesise to decrease the CCI by 10 points in the interventional group (minimal important difference, [40]). The primary endpoint is tested simultaneously using a two-sided *t* test in the subgroup of patients with a minimally invasive surgery and the subgroup of patients experiencing an open surgery. The ratio of minimally invasive to open is expected to be 1:1. Therefore, the overall two-sided significance level of α = 0.05 is adjusted by Bonferroni correction, yielding α = 0.025 for each of the two subgroups. Thus, to achieve a power of 80%, a sample size of *n* = 156 (78 per group) has to be recruited per subgroup with a total required sample size of the trial of *n* = 312 (156 per therapy group). To compensate for drop-outs and lost-to-follow-ups, a further 10% of patients will be randomised, leading to a total sample size of *n* = 348 (174 per group). Patients who die during the observation period receive the maximum number of 100 CCI points per definition [39, 40].

### Statistical analysis

For the examination of the primary endpoint “CCI within 30 days after the index operation”, the hypotheses to be assessed for each subgroup (minimally invasive or open) in the primary analysis are as follows: H0: μ1 = μ2 vs. H1: μ1 ≠ μ2, where μ1 and μ2 denote the mean CCI in the control and the intervention group respectively. The significance level is set to a two-sided α = 0.025 per subgroup test. Due to the stratified randomisation and relatively large number of centres in relation to the sample size, inclusion of centre as a random effect is recommended [51]. Therefore, the primary endpoint will be examined in the respective subgroup (minimally invasive or open) using a linear mixed model including centre as random intercept and group as fixed effect, which leads to equal or even increased power as compared to using a conventional two-sided *t* test [52].

The primary analysis will be conducted based on the full analysis set according to the intention-to-treat (ITT) principle and comprises all patients in the group they were randomised to. In the ITT analysis, missing data for the primary outcome variable will be replaced by using multiple imputation, which takes the covariates treatment group and centre into account by application of the fully conditional specification method [53]. The per protocol (PP) set consists of all patients treated per protocol; no missing data will be imputed. An additional mobility population consists of all patients who were randomised, had no re-operation within 30 days and had not been on the intensive or intermediate care ward for ≥ 4 days during the first postoperative week.

In general, for the full analysis set, all baseline values and secondary outcomes will be evaluated descriptively, and descriptive *p* values are reported together with 95% confidence intervals for the corresponding effects. Thereby, secondary endpoints will be evaluated descriptively using regression models including group as fixed effect and centre as random intercept as specified for the primary endpoint.

In further exploratory analyses, the association of variables with the primary and secondary outcomes will be assessed. In addition, subgroup analyses will be carried out. The safety analysis includes calculation of frequencies and rates of complications together with 95% confidence intervals. All analyses will be done using SAS version 9.4 or higher. The analyses will be described in further detail in the statistical analysis plan, which will be written before database closure.

It is planned to make all trial data publicly available after the publication of trial results.

### Risk of bias

In order to ensure that both treatment groups are well balanced for known and unknown confounders, the assignment to the two groups is based on randomisation. In order to document potential selection bias, all consecutive screened patients are listed in a screening list. To exclude the quality of surgery as a key factor for postoperative complications, randomisation is performed at the time of skin closure or later. In order to standardise outcome assessment, medical students are trained during a clinical investigator training (CIT) course in study-specific procedures as well as good clinical practice to reduce detection bias. The curriculum of the CIT has been described elsewhere [36]. Furthermore, obligatory online training material is available to all participating students. In addition, medical students are supported by surgeons experienced in clinical trials (clinician scientists) at their local sites. Blinding, however, is impractical in our study setting, as it would require ornate blinding methods, which contradict the pragmatic trial design. However, in order to counteract the risk of bias introduced by the unblinded study design, we have chosen a primary endpoint robust against unconscious or intentional influence, namely the Comprehensive Complication Index (CCI). The Dindo-Clavien complication classification, on which the CCI is based, is an objective outcome measure, as it grades complications according to their sequelae (e.g. death, re-operation, re-intervention, etc.) and can hardly be influenced by subjective impressions. However, this does not hold true for some of the secondary outcomes, e.g. patient-reported measures like pain. Furthermore, follow-up is limited to 30 days; thus, few missing values are expected. Since patients are still hospitalised for parts of this period, the attrition bias is expected to be low.

In addition, known courses for postoperative recovery will be meticulously recorded during follow-up in order to record potential confounders. These include the preoperative physical fitness (assessed via the DASI score), the impact of surgery (laparoscopic vs. open), the type of recovery programme, analgesic regime, duration of catheter, drain and tube placement and the degree of physiotherapy.

### Risk-benefit assessment

All patients will receive medical treatment including surgery as defined by their treating physicians (gold standard). No changes in medical or surgical therapy will occur due to the trial. However, all participating patients have an additional time burden of filling out the patient-reported outcome measures (QoR-15 questionnaire, EORTC QLQ-C30) and the time needed for the clinical trial visits. In addition, patients in the interventional group might have the added risk of ambulation-associated adverse events like burst abdomen, unintended falls and increased pain during mobilisation. However, evidence indicates that early and enhanced ambulation is beneficial rather than harmful for patients [22]. Consequently, all current guidelines recommend enhanced postoperative mobilisation [16, 17]. Thus, patients in the control group rather than the interventional group have an added risk for immobilisation-associated complications like deep vein thrombosis and postoperative pulmonary complications. However, whether or not FTs enhance postoperative ambulation following major abdominal surgery is unclear. Furthermore, it is unclear whether or not FTs are able to reduce immobilisation-associated adverse events. In summary, based on current evidence, clinical equipoise is given, which necessitates the current trial.

### Data management

An electronic case report form (eCRF) implemented in the REDCap™ system [54] will be used for data collection [55]. All protocol-required information collected during the trial must be entered by the investigator, medical student or designated representative in the eCRF in hospital. For health-related quality of life and patient-reported outcomes, the data must be entered directly by the patients in the eCRF. To this end, tablets or laptops are used. Alternatively, paper-based reported outcome questionnaires may be used and must then be entered by the investigator, medical student or designated representative in the eCRF. For follow-up visits, patients access the eCRF directly online. Data transmission is encrypted with Secure Sockets Layer (SSL) technology. The database server is located in a secure data centre and is protected by a firewall. The system provides an infrastructure to support user roles and rights. Only authorised users are able to enter or edit data, and access is restricted to data of the patients in the respective centre. All changes to data are logged with a computerised timestamp in an audit trail. All clinical data will be pseudonymised.

All protocol-required information collected during the trial must be entered by the investigator, medical student or designated representative in the eCRF. For health-related quality of life and patient-reported outcome data, patients may directly enter the data in the eCRF. Alternatively, paper-based reported outcome questionnaires must be entered by the investigator, medical student or designated representative in the eCRF. The completed eCRF must be reviewed and signed by the local investigator or by a designated sub-investigator.

In order to guarantee high quality of data, the completeness, validity and plausibility of data as defined in a data validation plan will be checked at the time of data entry and using validating programs, which will generate queries (centralised monitoring). The investigator, medical student or the designated representatives are obliged to clarify or explain the queries.

Data from the FTs are collected at each local site according to the manufacturers’ guidelines. Download from data of FTs takes place either at individual end of study or if the FT has to be exchanged for any reason (low battery, inconsistency between patient’s physical activity and FT step count). Raw data files from each patient are stored at the local sites according to the applicable local, national and international regulations. Raw data files are transferred via a secure upload server at the Institute of Medical Biometry and Informatics (IMBI) from the local sites to the IMBI.

All data collected (eCRF data and FT data) will be integrated in a statistical analysis system. During study conduct, database access will be granted to the data manager only. After database closure, access rights will be granted to the responsible biometricians as well. The data will be managed and analysed in accordance with the appropriate Standard Operating Procedures (SOPs) valid at the IMBI, Heidelberg.

## Discussion

Reducing postoperative complications in major abdominal surgery is one of the major challenges for the future. Despite innovations like minimally invasive surgery, postoperative complication rates of more than 30% are regularly reported [5–15]. Non-surgical complications including pulmonary and cardiovascular complications contribute a substantial part [13]. Mobilisation has been postulated to reduce postoperative morbidity and is part of all current ERAS guidelines; however, evidence to support this concept is low [18–21]. A number of studies have investigated mobilisation as one part of a “bundle of interventions” (as in ERAS programmes), frequently with favourable outcome [56, 57]. We hypothesise that patients in the interventional group have a significantly higher step count than patients in the control group. The size of the contribution of each individual treatment component within a care bundle, however, limits interpretation of findings. In line with this, the real effect of fast-track protocols on postoperative complications in minimally invasive surgery is controversial [10]. Interestingly, only 20–28% of patients were mobilised on the first postoperative day after liver surgery, despite predefined mobilisation targets [58–60]. Others reported that only a minority of patients achieved the preset mobilisation targets following colorectal cancer surgery, which could not be improved by the implementation of an ERAS pathway [61].

A systematic review investigating postoperative mobilisation identified only three RCTs and one observational study with poor methodological quality, including merely 225 patients [22]. None of these studies used postoperative complications as the primary outcome parameter, but looked at surrogate parameters like number of steps. Furthermore, none of the trials applied standardised outcome definitions for postoperative complications.

Importantly, the intervention known as “early and enhanced” mobilisation is not clearly defined, as studies use different postoperative mobilisation protocols. Thus, it is unknown whether postoperative “mobilisation targets” have any beneficial effect and, if so, how these targets should be defined. Hence, our pragmatic trial is a step towards a standardised mobilisation protocol in major abdominal surgery and adheres to high methodological standards. Future studies could then refine step goals in different clinical settings (e.g. laparoscopic vs. open surgery) and based on an individual’s personal fitness, eventually enabling evidence-based personalised mobilisation regimens.

Mobilisation is no trivial aspect in the postoperative course, as recent research suggests that enforcing early mobilisation targets requires substantial staff time. The lack of manpower is a main barrier to this practice [62]. Furthermore, many patients fail common ERAS mobilisation targets in major abdominal surgery [48].

FTs are becoming increasingly popular as measurement tools in clinical research [23]. Potential advantages of using FTs in postoperative surgical patients are (1) real-time continuous feedback; (2) objective, validated outcome measurement of physical activity including step counts rather than subjective, error-prone assessment by patients themselves or hospital staff; (3) reduction of staff time and manpower to enforce mobilisation targets, thereby (4) increasing the cost-effectiveness of mobilisation interventions, which has been identified as a main barrier to its implementation [62].

As with mobilisation, evidence to support FTs in the postoperative setting is limited. A major difficulty in comparing results of studies including wearable fitness devices is the heterogeneity of interventions and high risk of bias [63]. Studies analysing the association of early postoperative mobilisation and clinical outcome focus on step counts rather than patient-relevant outcome measures like complications [26, 28, 64]. Cook et al. and Daskivich et al. reported a prognostic relationship between the number of postoperative steps and length of stay [29, 65]. Wolk et al. performed a single-centre RCT in major abdominal surgery comparing the use of FTs plus a daily step goal vs. FTs with blinded display and no daily step goal (standard of care). The mean number of steps within the first 5 postoperative days (PODs) was increased after laparoscopic but not after open abdominal surgery [24]. Ni et al. performed an exploratory single-centre RCT in hepatic surgery using FTs as step count and enforced “early and enhanced” mobilisation by health-care team workers. They reported faster return of gastrointestinal function and a decreased length of hospital stay in favour of the mobilisation group, although postoperative complications were not significantly different between the groups [25]. Thus, the actual impact of feedback by wearable devices and health-care staff on physical activity remains uncertain.

The proposed trial has several strengths and limitations. Our trial addresses common limitations associated with the use of FTs. First, the accuracy of commercially available devices in the clinical setting is low (see e.g. [23, 66, 67]) owing to specificities of the postoperative period like shuffling gait. Second, concerns about data protection are inadequately addressed by commercial FTs. We created a secure digital environment (eCRF) which allows the storage and analysis of data according to European and national regulations. Third, most commercial FTs do not provide raw data, as is needed for unbiased trial analysis, but rather aggregated data [67]. By using devices especially made for clinical research and which provide raw data, our study circumvents these problems. Fourth, EXPELLIARMUS will evaluate mobilisation with rigorous methodology, using validated outcome measures. Accordingly, EXPELLIARMUS has a multicentre RCT design to test the efficacy of enhanced mobilisation and FT-based feedback. It will use the validated and internationally accepted CCI as the primary endpoint, thus focusing on the most patient-relevant outcome. Fifth, mobilisation is assessed by numerous functional parameters like the 6-MWT or ambulation > 10 min, as has been proposed before [22]. In addition, validated patient-reported outcome measures like the QoR-15 or EORTC QLQ-C30 as well as safety measures will be assessed to give a comprehensive picture of the potential risks and benefits of FT-based enhanced mobilisation. Finally, as a unique characteristic of our trial, the organisation, conduct and analysis will be done by a network of medical students with the support of experienced surgeons, clinician scientists and statisticians in a large research-based learning project. By planning, conducting and analysing a clinical trial, students will not only gain clinical knowledge and experience, but also scientific competencies. Projects to provide such research experiences are emerging and show that medical students can improve their clinical capabilities by contributing to student-initiated clinical trials [68, 69]. A student-led initiative in the UK has successfully performed a number of large observational studies in a national cohort following gastrointestinal surgery, demonstrating the feasibility of this concept [70, 71]. On an even larger scale, the EuroSurg collaborative demonstrated the feasibility of this concept across multiple European countries [72].

Despite successful student-led initiatives in other countries [73] and studies by the SIGMA network itself [37], this set-up could also be viewed as a major limitation. All previous studies were observational studies with a much simpler design, and it is unknown whether medical students can successfully conduct an RCT. In addition, it is unknown whether medical students assess and report postoperative complications with a quality comparable to that of trained clinician scientists. Another limitation of the trial is the unblinded trial design. Thus, we cannot exclude performance bias in both groups. However, the Dindo-Clavien classification, on which the CCI is based, grades postoperative complications according to their sequelae. Grade III (requiring endoscopic, radiologic or surgical intervention), IV (requiring intensive care treatment) and V (death) complications are objective and can hardly be influenced even in an unblinded study setting. However, grade I (any deviation from the normal postoperative course without the need for pharmacological treatment) to grade II (pharmacological intervention) complications are more prone to subjective interpretation and might be prone to bias in our study. However, grade I and II complications give only a few CCI points, and our outcome assessors (medical students) have no incentive to “fake” complications. As patients are aware of being part of a mobilisation trial, patients in the control group might be especially motivated to ambulate. Furthermore, as randomisation occurs on an individual level, we cannot exclude “contamination” of treatment groups. However, by using an objective, well-standardised primary outcome measure, we believe our results are robust against potential bias due to unblinding.

## Conclusions

EXPELLIARMUS will provide high-quality data on the efficacy of fitness tracker-based feedback following major abdominal surgery. Supported by surgeons and trained study staff, this large multicentre RCT will provide medical students with an opportunity to acquire scientific competencies and clinical research skills in a large research-based learning project.

## Trial status

The protocol is version 1.1. (5 March 2019). Recruitment started (first patient in) on 5 June 2019; 219 patients have been included in the trial (31 January 2020). The approximate date when recruitment will be completed is 31 May 2020.

## Data Availability

Data sharing is not applicable to this article, as no datasets were generated or analysed. The pseudonymised data of the EXPELLIARMUS study will be available to the participating clinical trials sites for further analyses if the planned post hoc analyses are performed in accordance with data protection laws and ICH-GCP guidelines. Anonymised data of the EXPELLIARMUS study may be available to other researchers if their planned analyses are performed in accordance with data protection laws and ICH-GCP guidelines.

## Abbreviations

6-MWT: 6-min walking test
ASA: American Society of Anesthesiologists
CCI: Comprehensive Complication Index
CHIR-*Net*: Clinical Trial Network of the German Surgical Society
CIT: Clinical investigator training
CRF: Case report form
DASI: Duke Activity Status Index
DRKS: Deutsches Register Klinischer Studien. The German Registry of Clinical Trials (WHO-compatible)
EORTC QLQ-C30: European Organisation for the Research and Treatment of Cancer Quality of Life Questionnaire C30
ERAS: Enhanced Recovery After Surgery
F/U: Follow-up
FT: Fitness tracker
GCP: Good clinical practice
GI-2: Gastrointestinal-2 (score)
HRQoL: Health-related quality of life
ITT: Intention-to-treat
NRS: Numeric rating scale
POD: Postoperative day
PP: Per protocol
QoR-15: Quality of Recovery 15 questionnaire
RCT: Randomised controlled trial
SIGMA: Student-Initiated German Medical Audit
